# Short-Term Effects of a Myofunctional Appliance on Atypical Swallowing and Lip Strength: A Prospective Study

**DOI:** 10.3390/jcm9082652

**Published:** 2020-08-15

**Authors:** Vincenzo Quinzi, Alessandro Nota, Eleonora Caggiati, Sabina Saccomanno, Giuseppe Marzo, Simona Tecco

**Affiliations:** 1 Department of Life, Health and Environmental Sciences, University of L’Aquila, 67100 L’Aquila, Italy; vincenzo.quinzi@univaq.it (V.Q.); eleonora.caggiati@gmail.com (E.C.); sabinasaccomanno@hotmail.it (S.S.); giuseppe.marzo@univaq.it (G.M.); 2 Department of Dentistry, Vita-Salute San Raffaele University, I.R.C.C.S. San Raffaele Hospital, 20132 Milan, Italy; nota.alessandro@hsr.it

**Keywords:** interceptive orthodontics, orthodontic removable appliance, myofunctional appliance, atypical swallowing, lip strength, orthodontics

## Abstract

Atypical swallowing needs treatment in order to eliminate harmful interferences of the tongue, which prevent the harmonious growth of the stomatognathic system. The purpose of this study was to assess the effects of a functional appliance on the presence of atypical swallowing, analyzing the lip strength and the altered facial mimics. The effects of a myofunctional appliance (the Froggy Mouth) were evaluated on 40 children (6 males; 24 females; mean age 9.6 ± 2.17) with atypical swallowing—with tongue thrust diagnosed by an expert orthodontist—before and during a 6 month treatment. Data were analyzed over time with a paired samples t-test for normally distributed data. After 6 months of treatment, 33 children out of 40 achieved clinical correction of atypical swallowing due to their good compliance, even at an early stage. Seven children showed low compliance and did not obtain any result. Lip strength in compliant subjects went from 190.30 ± 86.04 cN to 489.39 ± 123.36 cN (t = *p* < 0.001). Facial mimics improved in 28 out of 33 compliant subjects, and four children with the initial diagnosis of labial incompetence achieved correction. This observational study demonstrates the short-term efficacy of this myofunctional appliance in the treatment of atypical swallowing, achieving correction of the facial mimics and labial incompetence with a significant improvement of the lip strength.

## 1. Introduction

The physiological swallowing of the adult consists of positioning the tip of the tongue at the level of the incisive papilla with the dental arches in contact. A swallowing pattern that deviates from this is called atypical swallowing, a term that indicates a pattern characterized by tongue thrust between the dental arches during swallowing [1,2,3]. In this case, treatment is needed in order to eliminate harmful interferences of the tongue, which prevent the harmonious growth of the stomatognathic system [4].

The first rudimentary swallowing acts begin from the 11th week of gestational age, but it is only towards the 16th week of intrauterine life that the tongue assumes a position similar to that of the infant during breastfeeding [1], with a pattern that is normal during the prenatal period (swallowing the amniotic fluid), the neonatal period (sucking the nipple) and the first years of life, and has a paracortical origin, controlled by the facial nerves [2]. After that period, the swallowing pattern progressively adapts to feeding methods (from sucking to the use of a spoon), to neuromuscular development, as well as to the development from mixed to definitive dentition [1,3,5]. The transition from infantile to adult swallowing takes place between 3 and 7 years of age. When the infantile swallowing persists beyond the age where the adult type should have appeared physiologically (about 7 years), one can speak of atypical swallowing [6]. The main causes of atypical swallowing (infantile swallowing) are related to incorrect eating habits (prolonged breastfeeding, delayed weaning, liquid diet, etc.), poor oral habits (thumb sucking, prolonged use of the pacifier, oral breathing, etc.) or pathological causes (hypertrophy of the pharyngeal and/or palatine tonsils, hypertrophy of the turbinate, macroglossia, etc.) [7,8].

From a clinical point of view, atypical swallowing involves lingual interposition, contraction of the mental muscle, interposition of the lower lip between dental arches, and consensual movements of the head and neck. It can have consequences for breathing, chewing, speech and posture [9,10,11], as was also observed for other stomatognathic disorders, such as temporomandibular disorders (TMD) [12,13,14,15,16]. Among these complications, there is also an evident alteration of the facial profile and mimicry, accompanied by hypertonia of the chin and hypotonia of the orbicularis oris muscle [4,17]. In particular, the lip strength is related to altered jaw movements, for example during mandibular protrusion [17] and is involved in labial incompetence, while it is also critical in maintaining equilibrium in anterior teeth position [18]. It also seems that lip strength deficits could lead to significant impairment of daily function and facial growth, because all the facial muscles of the splanchnocranium act in synchrony, and during chewing all the perioral facial muscles are activated, at the same time as the lip [19]. Consequently, the treatment of atypical swallowing is necessary not only to eliminate harmful interferences of tongue thrusting, but also to allow the harmonious growth of the stomatognathic system [4,8,17] and the whole face, potentially preserving/achieving physiological lip strength.

Various therapies have been proposed to treat atypical swallowing. Among these, functional devices such as the Bionator [20], the Fraenkel [21], eruption guidance appliances, lingual spurs [22], fixed grids [22], as well as the speech therapy treatment [23], and myofunctional therapy (MFT) [9,24]. A new appliance, the Froggy Mouth (FM), was first proposed by Dr. Patrick Fellus in 2016 [3,25]. It is a small removable device made of a flexible material, thermoplastic elastomer (TPE), which, differently from other devices, is not placed inside the mouth but between the lips and inhibits sucking-swallowing, as well as stimulating the contraction of the lip to keep the device stable [25]. Thus, it is considered a myofunctional appliance that, when positioned between the lips, prevents bilabial contact, stimulates muscular training, and forces the tongue in a correct position, aiming to induce a new swallowing pattern. FM can be prescribed to very young children as it is not necessary to take impressions/scans to produce the device.

The purpose of this study was to assess the effects of FM therapy on the presence of atypical swallowing and lip strength.

## 2. Experimental Section

The present prospective study was carried out on a sample of patients from a private practice in Rome (Italy). The STROBE checklist for observational studies was used to report data. A total sample of 48 children, aged 5–13 years (24 females, 16 males; mean age 9.30 ± 2.17 years), with atypical swallowing with tongue thrust diagnosed by an expert orthodontist, were initially enrolled in the study. Tongue thrust was defined as the protrusion of the tongue between upper and lower incisors or cuspids during swallowing [26].

**Figure 1:**
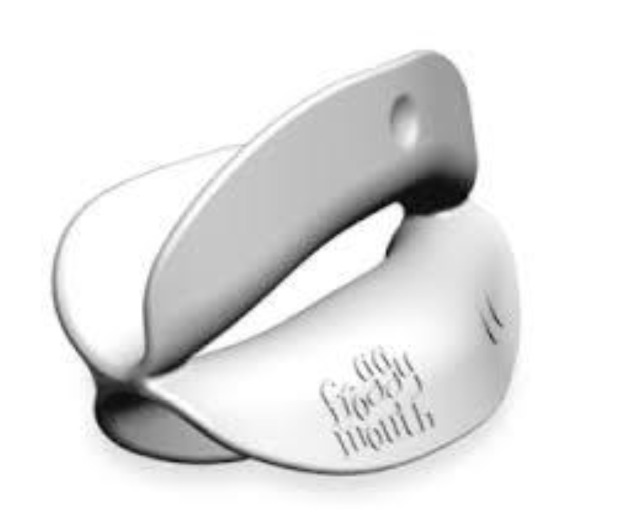
Froggy Mouth (FM) (Micerium, Genoa, Italy).

The protocol was in accordance with the Declaration of Helsinki and it was ethically approved by the University of L’Aquila (document DR 206/2013). Myofunctional treatment with Froggy Mouth (Micerium, Genoa, Italy) (Figure 1) was proposed to all of the enrolled patients.

**Figure 2:**
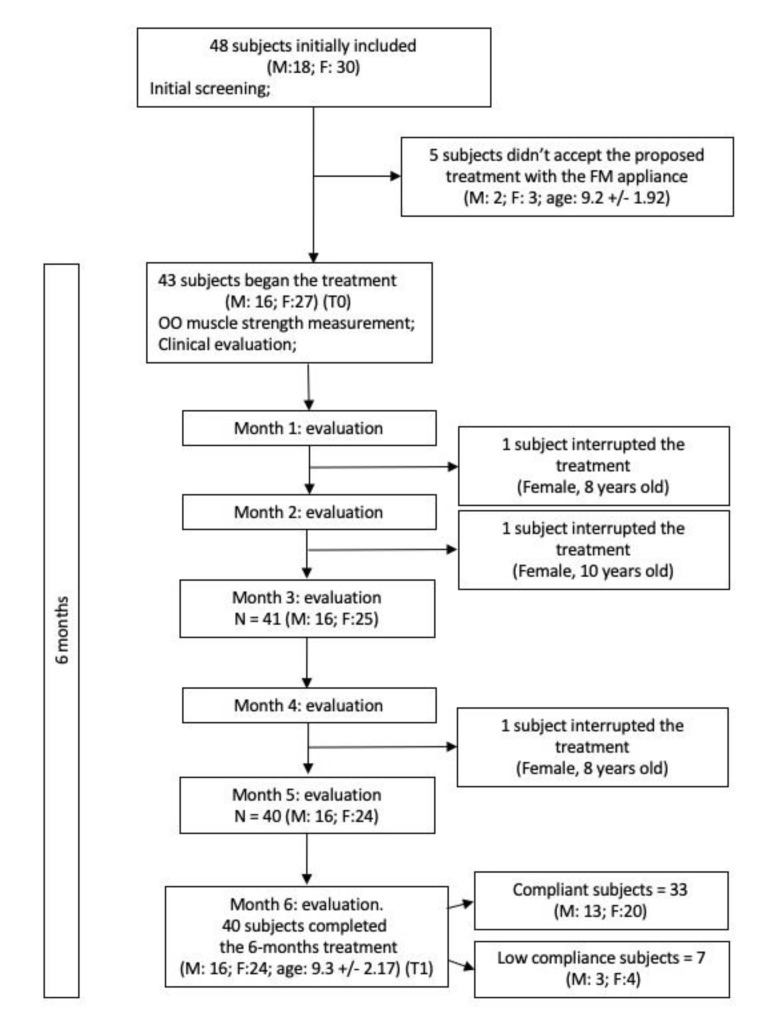
Study flow chart.

Five subjects (3 females, 2 males, mean age 9.20 ± 1.92 years) did not accept the treatment plan and three children (3 females; mean age 8.67 ± 1.15 years) did not complete the protocol. Therefore, a final sample of 40 children with atypical swallowing was included in this study. The flow chart of the study is represented in Figure 2.

**Figure 3:**
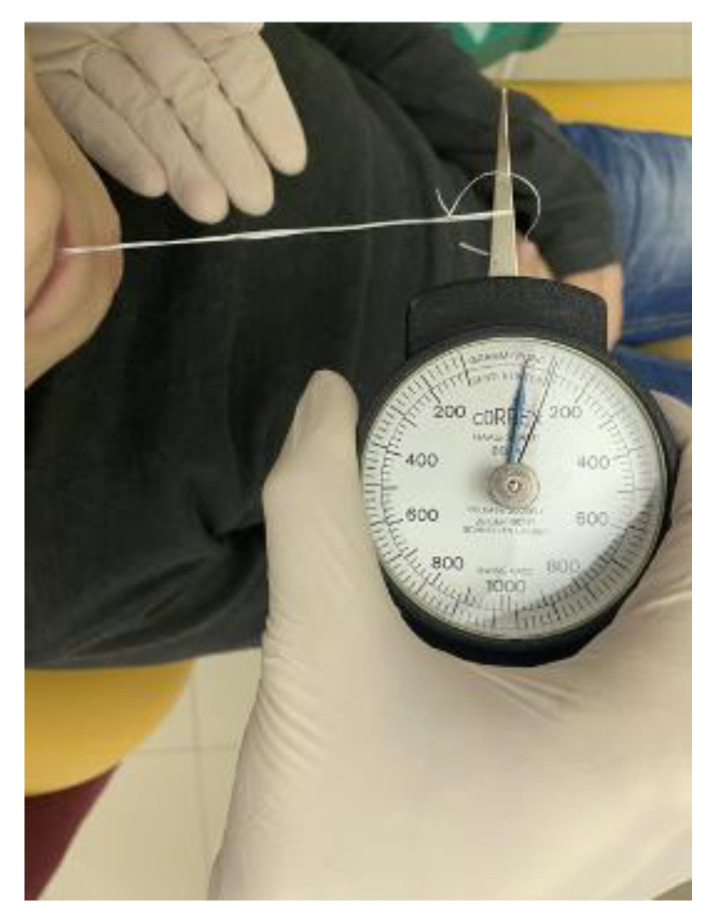
Strength measurement of the orbicularis muscle. A dynamometer was used in a standardized way: the length of the thread was 40 cm; the thread was folded in half, with a button on one end, and a knot on the other.

The FM appliance was prescribed in the appropriate size (small, medium, large): small for children between 3 and 7 years old, medium for children between 7 and 12 years old, large for children over 12 years old and adults. According to the manufacturer’s instructions, the patients were asked to wear FM for 15 min a day for a period of 6 months, while sitting in an upright position, watching television (positioned at a minimum distance of 2 m) or during recreational activity. Every month, each patient underwent a clinical and lip strength evaluation, as well as the recording of a video clip while sitting in an upright position. The evaluation of the swallowing function was always performed by a single clinician (the author E.C.) with experience in the area, as previously reported in literature [26,27]. Lip strength detection was standardized and performed on each subject after a proper training session by the same operator. The child was asked to sit on the dental chair with the head in a natural position, leaning against the headrest, keeping anterior to the teeth and posterior to the lips (without suction) a button connected with a 40-cm-long piece of thread folded in half and tied with a final knot. A dynamometer (Correx, Haag-Streit Diagnostics, Koeniz, Switzerland) was then connected to the folded thread and a continuous increasing force, ideally parallel to the floor, was applied by the examiner directly, away from the patient’s mouth, until the lips were unable to hold onto the button, reaching the maximum resistance of the orbicularis muscle. This lip strength measure system was previously used by Berggren et al. [28] (Figure 3).

One repetition of the measurement was performed in order to confirm the correct measurement of the maximum strength. The recorded force was then reported in the data sheet. A preliminary study was performed to evaluate the intra-observer repeatability of the clinical evaluation and lip strength. The same operator was asked to analyze a sample of 10 subjects twice, and an accordance with 100% of the clinical data was proved, also there was not any statistically significant difference between the first and the second evaluations regarding lip strength.

Due to the absence of a previous similar study, the study protocol initially hypothesized a total number of 50 recruited subjects, considering a high probability that young subjects might miss one of the scheduled monthly appointments or show low compliance with the treatment, planning a minimum sample size calculation to be performed after achieving full data on 20 subjects. On the basis of preliminary data calculated on the first 20 subjects of the present study, the minimum required sample size was estimated, and because of the considerable variation of the parameters between before the treatment (t0) and after six months of treatment (t1), the minimum sample to achieve 95% of power with 0.05 alpha error was 5 subjects. Considering that a total of 48 subjects had already started the protocol at that time, the study was completed with a total of 40 subjects (33 with proper compliance) anyway, achieving a representative sample with an actual sample power higher than 99%.

The primary outcome (lip strength) was treated as a continuous variable, in centiNewton (cN), and resulted in normal distribution according to the Kolmogorov–Smirnov test. Thus, a descriptive statistical analysis was performed, providing mean values and standard deviations at t0 (before the treatment) and t1 (after six months of treatment). Over time, differences were tested with a paired samples t-test for normally distributed data. In addition, data from clinical evaluations of swallowing were recorded as nominal variables dichotomized as corrected/uncorrected swallowing patterns, as previously reported in literature [27] and then described as frequencies of patients with corrected/uncorrected swallowing for each month of follow-up. For each test, *p*-value was set at 0.05 level. Statistical analyses were performed with PSPP software, version 1.2.0. (GNU project, Free Software Foundation, Boston, MA, USA).

## 3. Results

**Figure 4:**
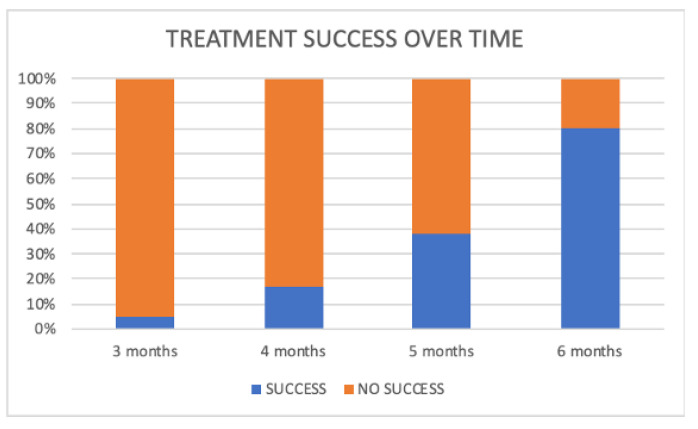
Increase in the percentage of subjects with treatment success vs. no success recorded over time in the whole sample (*n* = 40).

This observational study revealed that after six months 82.5% of the subjects (33 out of 40 children) showed good compliance and all of them achieved the clinical result (corrected swallowing pattern). Data sheet are available as supplementary material (Supplementary file 1). Among them, 2 children (5% of the whole sample) obtained an early correction after only 3 months, 5 children (12.5% of the whole sample) after 4 months, 11 children (27.5% of the whole sample) after 5 months and 15 children (37.5% of the whole sample) after 6 months (Figure 4).

An unsatisfactory compliance was reported in 17.5% of the whole sample (7 children out of 40) that did not scrupulously adhere to the prescribed protocol and neglected the appliance; consequently, they did not achieve any result. Thus, in compliant subjects there was 100% of treatment success.

**Figure 5:**
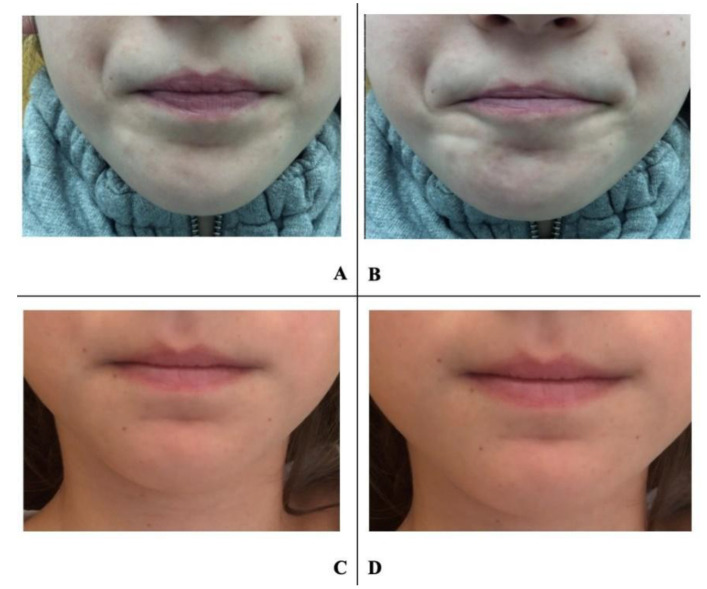
An 8-year-old patient. (**A–B**) before treatment; (**C–D**) after 6 months of treatment; (**A**) rest position, before treatment; (**B**) during swallowing, before treatment; (**C**) rest position, after treatment; (**D**) during swallowing, after treatment.

Clinical observations showed that most patients evolved in a progressive and constant way over the observational period, finally achieving the result. An example of treatment success on facial mimics is represented in Figure 5, in which photos of lip mimics during deglutition of an 8-year-old female are presented.

**Figure 6:**
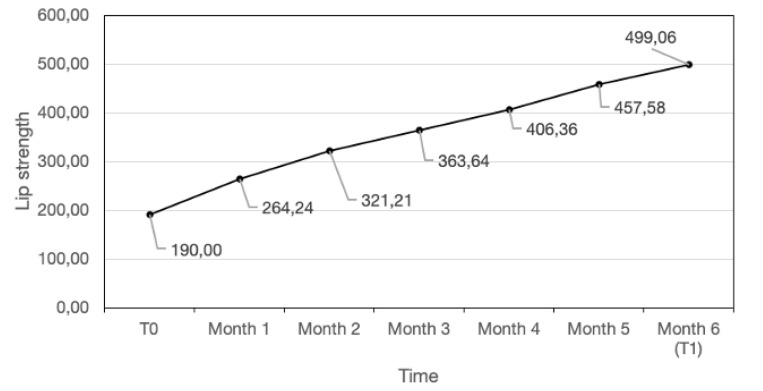
Lip strength (measured in centiNewton) over the study period in compliant subjects.

In addition to the successful treatment in 33 subjects out of 40, clinical analyses also showed a normalization of facial mimicry in 84.84% of the compliant patients (28 out of 33), while in five patients, despite the normalization of swallowing, a residual alteration of the facial mimicry was still visible at t1 (after 6 months), although considerably reduced compared with t0 and with improvement of the lip strength. In addition, all of the four compliant children with labial incompetence showed correction after treatment. No side effects were reported. None of the seven non-compliant children attributed their absence of compliance to problems caused by FM. With regard to maximum lip strength, a statistically significant mean increase of 257% was observed in compliant children (*p* < 0.0001) from t0 to t1, as represented in Table 1 and Figure 6.

## 4. Discussion

This observational study aimed to detect the effects of a myofunctional appliance after a 6 month treatment on the swallowing pattern and maximum lip strength in children affected by atypical swallowing. Atypical swallowing was detected clinically, through the observation of facial mimicry. The lip strength measurements objectivized the treatment success in terms of muscular changes associated with the swallowing pattern. To the best of the authors’ knowledge, this is the first study that analyzed the efficacy of this type of appliance (Froggy Mouth) in correcting atypical swallowing pattern and lip strength.

The present data evidenced a relevant progressive improvement of swallowing pattern and lip strength due to the treatment, with the achievement of a corrected pattern in 82.5% of the whole sample and in 100% of the compliant subjects. The 17.5% of the sample that showed a lack of compliance did not achieve treatment success. On the other hand, among compliant children there was even an early correction of the swallowing pattern: in two children after the third month, and in five children after the fourth month. These findings confirm the efficacy of this appliance in correcting atypical swallowing. Although FM requires lower compliance (only 15 min/day) [1,2,3] compared with other removable devices, there was a considerable percentage of non-compliant subjects, although none of them complained of any side or unwanted effects during the treatment. Low compliance could be related to the group’s mean age or other related variables, including their parent’s acceptance of the orthodontic treatment. With the FM appliance, the need for compliance is very low compared with other devices. However, at this age, children that are very busy because of school or sports engagements usually show poor compliance with removable orthodontic devices.

The main advantage of a mobile appliance is that it does not cause any hindrance to oral hygiene, while both vestibular [29,30] and lingual [31] fixed appliances are shown to alter the oral environment during treatment [32,33].

Among the compliant children, other clinical effects of the treatment were reported through facial mimics and labial incompetence. In particular, it was effective in normalizing facial mimics in 84.84% of the compliant subjects, as only five patients did not achieve a complete correction of facial mimics. This datum suggests that in some cases it could be advisable to extend the treatment time beyond 6 months, as further improvement of the results could be auspicated with a longer treatment. Therefore, clinical studies with a longer treatment duration should be performed to confirm this hypothesis. In addition, all the compliant children with labial incompetence (four subjects) achieved a correction of labial posture in addition to swallowing correction. The data on labial incompetence are thus very promising and higher than what is reported in literature for other treatment alternatives; for example, Giuca et al. reported an improvement of labial incompetence in only 14 children out of 35 (37%) after speech therapy [23].

In literature other appliances were used for the correction of atypical swallowing, such as the Fraenkel appliance, the Bionator appliance, lingual spurs, and eruption guidance appliances. They seem to achieve clinically valid results but require a greater compliance by the patient, involve difficulties in daily life, for example during phonation, can create intraoral injuries, and ultimately they negatively affect aesthetics. In addition, no longitudinal evaluations exist in literature about the use of these devices against atypical swallowing, except for the Bionator [23]. Speech therapy also seems like an effective treatment [23], but requires a long treatment time, as well as higher patient compliance requirements. Literature reports that the most used speech therapy methods reached 47% of corrected cases in a group of 57 children (aged 5 to 13, mean age 8.2) with atypical swallowing diagnosis, after almost six months of treatment [23]. Furthermore, speech therapy treatment is not effective in children who are too young, around 3–4 years of age, while, on the contrary, labio-therapy can be effective [3]. Myofunctional treatment was also a successful treatment alternative in a recent study by Begnoni et al., 2020 [9] which, similarly to the present study, showed 100% success in correcting atypical swallowing in compliant subjects, achieving the result after 10 weekly 45 min sessions with a phonetician and daily home exercises. In that study, a considerably faster correction of atypical swallowing was achieved compared with the present study, but it should be considered that the remarkably higher time, cost and treatment complexity could impact the compliance of very young children, while the treatment performed by Begnoni et al. was directed at adolescents and young adults, showing only about 10% of poor compliance [4,9].

Considering these observations, it seems that the FM appliance has many advantages compared with other devices/treatments: ease of use, efficacy on labial incompetence often associated with atypical swallowing, efficacy on facial mimics, no need for intraoral traditional or digital impressions, easy patient monitoring through telemedicine consults, reduced required daily usage time, fast achievement of results, reduced financial cost, no need for manual parent intervention during the treatment. Most of these could identify FM as a low compliance appliance.

The present study demonstrates a statistically significant increase in lip strength during the treatment (*p* < 0.001), suggesting a functional training of muscle fibers by the appliance, that can explain the observed clinical success. A previous study by Bigenzahn et al. [24] similarly observed the efficacy of MFT (often associated with orthodontic treatment) in increasing lip strength in a sample of subjects affected by orofacial dysfunctions affecting speech such as atypical swallowing, but their findings are not directly comparable with the present study mainly because of a different subject selection. However, in agreement with the present study, they also observed a doubled lip strength after treatment. It must be considered that a statistically significant increase in lip strength was also observed in the sub-sample of patients with low compliance who did not achieve swallowing correction; in fact, they expressed a statistically significant increase in lip strength. Thus, considering this favorable data, it is probable that these subjects could achieve clinical success with proper compliance and/or a longer treatment duration.

### Limitations of the Study

The present study has some limits, like the absence of an untreated control group, an observation time limited to 6 months and the absence of a mid–long-term follow-up with data at 12–24 months after the end of the treatment, to verify the stability of its results.

## 5. Conclusions

This observational study demonstrates the short-term efficacy of FM in the treatment of atypical swallowing, with a significant increase in lip strength and an associated correction of labial incompetence, when present. Furthermore, the absence of side or unwanted effects was reported. The clinician could trust in a correction of atypical swallowing after 6 months of FM therapy if proper compliance is achieved by motivating the child to wear the appliance for at least 15 min/day.

Further investigation should involve control groups and the evaluation of a longer treatment time and mid–long-term results after the end of the treatment, to verify the stability of its results.

## Figures and Tables

**Table 1:** Lip strength (measured in centiNewton) at t0 and t1, and evaluation of t0–t1 differences with paired sample’ *t*-test, in compliant children (*n* = 33) and low-compliant children (*n* = 7).

	Lip Strength	Lip Strength	t0–t1	Paired Sample’ *t* Test (*p*)
	at t0	at t1	Difference	
	(centiNewton)	(centiNewton)	Mean ± SD	
	Mean ± SD	Mean ± SD		
Compliant Children (*n* = 33)	190.30 ± 86.04	489.39 ± 123.36	−299.09 ± 128.63	−12.23 (*p* < 0.001)
Low compliant Children (*n* = 7)	231.43 ± 144.62	408.57 ± 91.55	177.14 ± 140.20	2.73 (*p* = 0.015)

## Supplementary Materials

The following are available online at https://www.mdpi.com/2077-0383/9/8/2652/s1, Supplementary file 1: Data sheet.
